# Pseudogenes transcribed in breast invasive carcinoma show subtype-specific expression and ceRNA potential

**DOI:** 10.1186/s12864-015-1227-8

**Published:** 2015-02-22

**Authors:** Joshua D Welch, Jeanette Baran-Gale, Charles M Perou, Praveen Sethupathy, Jan F Prins

**Affiliations:** Curriculum in Bioinformatics and Computational Biology, The University of North Carolina at Chapel Hill, Chapel Hill, NC 27599 USA; Department of Computer Science, The University of North Carolina at Chapel Hill, Chapel Hill, NC 27599 USA; Department of Genetics, The University of North Carolina at Chapel Hill, Chapel Hill, NC 27599 USA; Lineberger Comprehensive Cancer Center, The University of North Carolina at Chapel Hill, Chapel Hill, NC 27599 USA

## Abstract

**Background:**

Recent studies have shown that some pseudogenes are transcribed and contribute to cancer when dysregulated. In particular, pseudogene transcripts can function as competing endogenous RNAs (ceRNAs). The high similarity of gene and pseudogene nucleotide sequence has hindered experimental investigation of these mechanisms using RNA-seq. Furthermore, previous studies of pseudogenes in breast cancer have not integrated miRNA expression data in order to perform large-scale analysis of ceRNA potential. Thus, knowledge of both pseudogene ceRNA function and the role of pseudogene expression in cancer are restricted to isolated examples.

**Results:**

To investigate whether transcribed pseudogenes play a pervasive regulatory role in cancer, we developed a novel bioinformatic method for measuring pseudogene transcription from RNA-seq data. We applied this method to 819 breast cancer samples from The Cancer Genome Atlas (TCGA) project. We then clustered the samples using pseudogene expression levels and integrated sample-paired pseudogene, gene and miRNA expression data with miRNA target prediction to determine whether more pseudogenes have ceRNA potential than expected by chance.

**Conclusions:**

Our analysis identifies with high confidence a set of 440 pseudogenes that are transcribed in breast cancer tissue. Of this set, 309 pseudogenes exhibit significant differential expression among breast cancer subtypes. Hierarchical clustering using only pseudogene expression levels accurately separates tumor samples from normal samples and discriminates the Basal subtype from the Luminal and Her2 subtypes. Correlation analysis shows more positively correlated pseudogene-parent gene pairs and negatively correlated pseudogene-miRNA pairs than expected by chance. Furthermore, 177 transcribed pseudogenes possess binding sites for co-expressed miRNAs that are also predicted to target their parent genes. Taken together, these results increase the catalog of putative pseudogene ceRNAs and suggest that pseudogene transcription in breast cancer may play a larger role than previously appreciated.

**Electronic supplementary material:**

The online version of this article (doi:10.1186/s12864-015-1227-8) contains supplementary material, which is available to authorized users.

## Background

Pseudogenes are genomic sequences sharing considerable sequence identity with protein-coding genes yet possessing features such as premature stop codons, deletions/insertions, or frameshift mutations that prevent them from producing functional proteins. There are three classes of pseudogenes: *processed*, *duplicated*, and *unitary*. A processed pseudogene lacks introns, resembling a spliced transcript that was inserted into the genome. A duplicated pseudogene is essentially a partial or complete copy of a protein-coding gene, including introns and sometimes even upstream regulatory elements. Thus, for any processed or duplicated pseudogene, there is an associated protein-coding gene called its parent gene that is highly similar in sequence. The third type of pseudogene is the unitary pseudogene, which arises when a protein-coding gene loses its coding potential through the accumulation of mutations. Unitary pseudogenes therefore do not have parent genes.

According to the GENCODE pseudogene annotations (v.17), there are nearly 15,000 human pseudogenes. Since their discovery in 1977, pseudogenes have generally been considered “biologically inconsequential” and non-functional [1]. Therefore, the discovery that a number of pseudogenes, such as *PTENP1* [2], are transcribed was somewhat surprising. The ENCODE project recently performed a survey of publicly available expression data to identify transcribed pseudogenes, and found over 800 pseudogenes with strong evidence of transcription [3]. These transcribed pseudogenes showed both tissue-specific and constitutive expression profiles. In addition, many of the pseudogenes not found to be transcribed by ENCODE possessed properties indicative of transcription potential, including open chromatin, histone modifications that indicate transcriptional activity, transcription factor binding, and RNA polymerase II occupancy. Another recent study found evidence for over 2000 expressed pseudogenes in 13 different cancer and normal tissue types [4].

Although some pseudogenes are transcribed, this fact does not necessarily imply that pseudogene transcripts perform biologically important functions. However, recent research has revealed several mechanisms by which pseudogenes regulate gene expression. For example, in snail neurons, translation of the neural nitric oxide synthase mRNA is blocked by an antisense pseudogene transcript that binds to the mRNA [5]. Pseudogenes in mouse can form double-stranded RNA by base-pairing with their corresponding protein-coding genes and generate siRNAs to silence the expression of these genes [6]. Pseudogenes may also compete with mRNAs for transcript stability factors, as in the case of the human *HMGA1-p* pseudogene [7].

The most recent function identified for pseudogenes is post-transcriptional regulation of mRNA levels by competing for miRNAs. This mechanism was first discovered in animals when it was shown that two human pseudogenes, *PTENP1* and *KRASP1*, are transcribed and harbor miRNA response elements (MREs) for some of the same miRNAs that target their corresponding protein-coding genes, *PTEN* and *KRAS*, respectively [8]. By binding and sequestering miRNAs that would otherwise bind and regulate *PTEN* or *KRAS*, the corresponding pseudogenes free the protein-coding genes from miRNA target repression. Thus, if the pseudogene is transcribed at a low level, more miRNAs will be able to target the parent gene transcripts, whereas an increase in pseudogene transcription will cause fewer miRNAs to target the parent gene. In this way, pseudogene RNA can compete with the parent gene RNA for miRNAs and thereby influence gene expression. This mechanism of regulation was first characterized in plants, where it was termed “target mimicry” [9]. Competition for miRNAs had also been used to create exogenous “miRNA sponges” containing specific MREs designed to soak up micro-ribonucleoprotein complexes and de-repress natural miRNA targets [10]. Salmena et al. coined the term *competing endogenous RNA* (ceRNA) to describe the function of *PTENP1* and *KRASP1* [11]. In theory, any type of RNA molecule, including mRNA, transcribed pseudogenes, and long non-coding RNA (lncRNA), can function as a ceRNA, provided the molecule shares at least one MRE with another RNA [12]. A number of ceRNAs have been identified since the initial discovery of *PTENP1* and *KRASP1*, including mRNAs [13-15], and lncRNAs [16]. Non-coding transcripts may serve as more effective ceRNAs than mRNAs, since they are substrates for miRNA binding but are not translated. The absence of bound ribosomes on a non-coding transcript allows miRNAs to bind freely along the entire transcript rather than primarily in the regions that are outside the ribosome footprint as on mRNAs [17]. Transcribed pseudogenes are especially strong ceRNA candidates because pseudogenes are identified by alignment with protein-coding genes, so by definition, they possess strong sequence similarity with their corresponding parent genes. This suggests that pseudogenes are likely to share MREs with their parent protein-coding genes. In fact, the sequence similarity between the *PTEN* coding gene and the *PTENP1* pseudogene was one of the initial observations that led to the discovery of the ceRNA function of the *PTENP1* pseudogene [8].

Interestingly, several transcribed pseudogenes play a key role in the development of cancer. *PTENP1*, *KRASP1*, and *OCT4-pg4* are known to promote tumor progression through their roles as ceRNAs [8,18]. The pseudogenes *SUMO1P3* [19], *ATP8A2-Ψ* [4], and *Nanog-p8* [20] have each been shown to enable cancer progression, but the mechanisms by which they do this are unknown. *Ψ-PPM1K* was shown to suppress oncogenic cell growth in hepatocellular carcinoma by generating endogenous siRNAs [21]. *ATP8A2-Ψ* is an especially interesting case, because it is the first published example of a pseudogene that is differentially expressed among cancer subtypes, showing high expression in breast cancer samples with luminal histology but very little expression in basal samples [4]. Also, *ATP8A2-Ψ* was shown to induce tumor progression when overexpressed in breast cancer cell lines [4].

Recently, a survey of RNA-seq data from The Cancer Genome Atlas project spanning seven cancer types showed that pseudogenes can be used to classify cancer samples into clinically relevant subtypes [22]. In particular, this study found that pseudogene expression alone separates endometrial cancer samples into groups corresponding to the major histological subtypes. Another interesting result from this study is that pseudogene-defined subtypes in kidney cancer show different patient survival rates. In addition, 547 pseudogenes with subtype-specific expression in breast cancer were identified. Finally, using miRNA expression data in conjunction with gene and pseudogene expression levels, they identified 38 pseudogenes with potential to function as ceRNAs in kidney cancer.

The pseudogenes that have been shown to participate in ceRNA interactions or play a role in cancer certainly represent provocative examples. However, the difficulty of reliably quantifying pseudogene expression and the lack of suitable datasets have hindered attempts to study these phenomena on a large scale. Therefore, it is not known whether pseudogenes like *PTENP1* and *ATP8A2-Ψ* represent a few anomalous cases or point to a pervasive regulatory mechanism.

To begin to address this open and important question, we performed an investigation of the expression, subtype specificity, and ceRNA potential of transcribed pseudogenes in breast cancer using data from The Cancer Genome Atlas project (TCGA). The data include RNA-seq results for a total of 819 tumor and adjacent normal samples, along with sample-paired small RNA-seq. The dataset contains a representative sampling of breast cancer subtype, including 123 samples from the basal subtype, 60 her2 samples, 371 luminal A samples, and 170 luminal B samples. To the best of our knowledge, this study is the first to make use of sample-paired pseudogene and miRNA expression data to investigate the ceRNA mechanism in breast cancer.

## Results

### Reliable quantification of pseudogene expression

Reliable quantification of pseudogene expression remains a challenging problem for a number of reasons. First, since parent genes and pseudogenes are highly similar in nucleotide sequence, short RNA-seq reads derived from one may align equally well to the other one. Such reads are fundamentally ambiguous in terms of their origin. Second, some reads may have *nearly* identical alignment to locations in the gene and pseudogene, and their mapping is often determined by the location with the least error in alignment. However, this strategy is unreliable in the presence of subject-specific variation with respect to the reference genome, or in the event of base call errors during sequencing, since these can result in an incorrect assignment of the read. Third, some aligners may follow a parsimony strategy in which a “simple” alignment is preferred to a complex (e.g. spliced) alignment. In the case of a processed pseudogene that lacks splices, this approach may erroneously bias the alignments to the pseudogene rather than the parent gene. Finally, in some cases, aligners report only a subset of possible alignments as a result of the heuristics used. For all of these reasons, studies of gene and pseudogene expression using existing tools are likely inaccurate without additional considerations.

**Figure 1:**
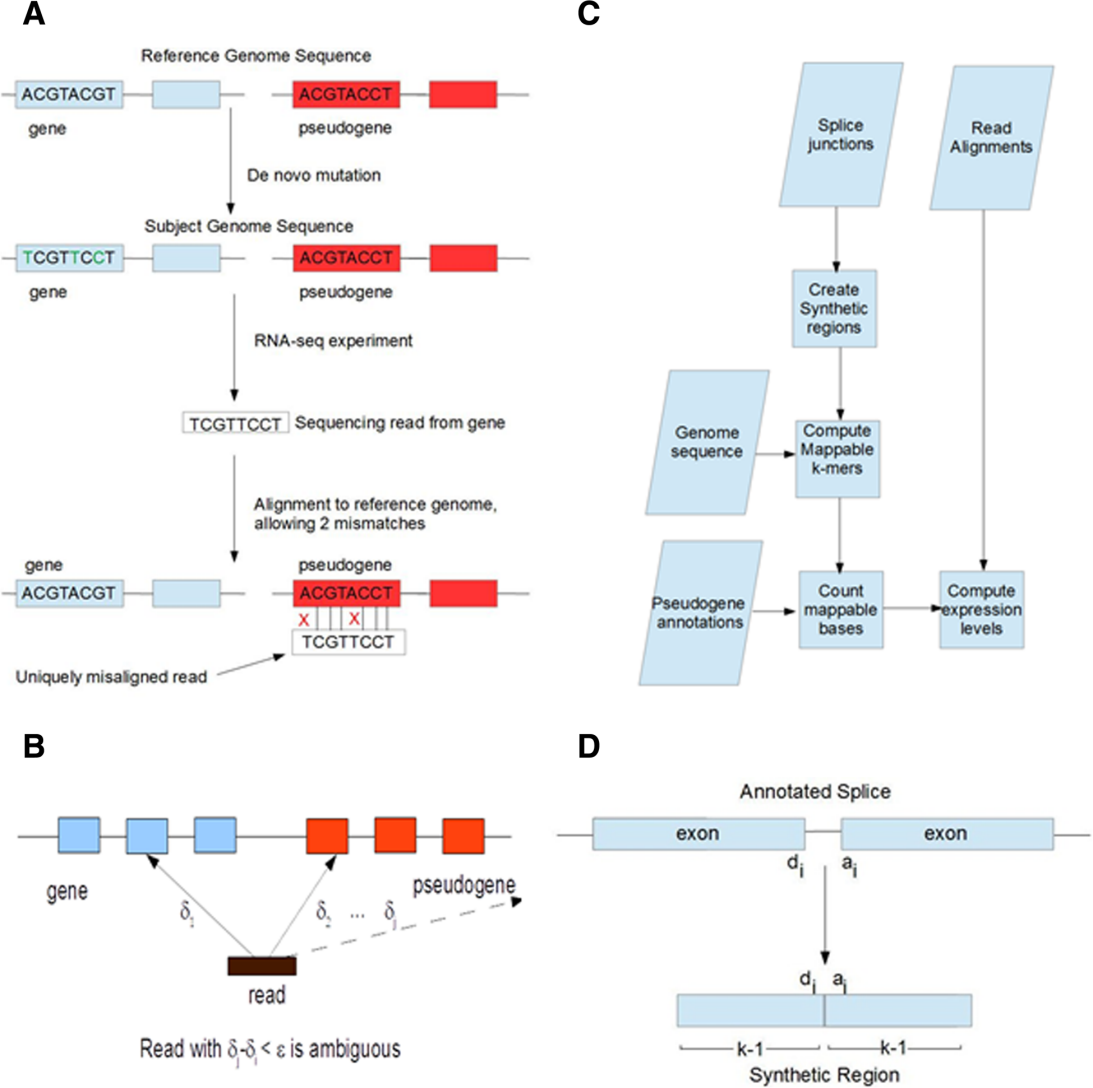
**Reliable quantification of pseudogene expression. (A)** Example showing that even an ideal aligner may produce uniquely misaligned reads in the presence of mutations and read errors if alignments to unmappable regions are considered trustworthy. In this example, the gene and pseudogene differ in one nucleotide so the regions are not identical. Now the gene in the subject genome being sequenced has undergone mutation so it differs from the reference genome in 3 positions. RNA-seq produces reads from this gene reflecting the mutations in the subject genome. If the reads are then mapped back to the genome allowing 2 mismatches, they map only to a pseudogene of the gene that produced the reads. The problem arises because the sequences of the gene and pseudogene are sufficiently similar that unique misalignment cannot be ruled out. **(B)** If a read has at least two alignments that are at distance δ_1_ and δ_2_ from the reference genome, respectively, then the true position of the read should be considered ambiguous unless |δ_1_-δ_2_| > ε for some integer safety margin ε > 0. **(C)** Pipeline for computing RPKUM expression levels for pseudogenes. **(D)** “Synthetic regions” around splice junctions are used to extend mappability to the transcriptome. A synthetic region is constructed by concatenating *k*–1 nucleotides from the donor and acceptor exons on either side of a splice junction. Any *k*-mer that crosses the splice junction thus occurs in the synthetic region.

A first approach to reliably studying pseudogene expression is to consider only the reads that are assigned to a single location by an aligner. However, the above confounding factors can result in reads that are uniquely aligned to the wrong positions (Figure 1A). Any conclusions drawn from such reads in downstream analyses will be unreliable. One approach to addressing this problem is to identify and discard from the analysis reads that map to regions in the genome that are especially sensitive to these confounding factors. We have adopted this approach using the concept of transcriptome mappability, which we describe below.

Our approach for computing transcriptome mappability builds on the notion of genomic mappability. Mappability is a measure of the inherent distinctiveness of a genomic region; the more frequently a genomic region occurs, the less mappable it is. Although mappability can be defined as a continuous quantity (the reciprocal of k-mer frequencies, for example, as in [23]), it is generally not very useful to know the degree to which a region is unmappable. If a k-mer occurs more than once in the genome, a read aligned there will be ambiguous. For this reason, we compute mappability as a discrete quantity –that is, a region is either mappable (unambiguous) or not mappable (ambiguous). Our notion of mappability also includes a “safety margin”, so that a mappable region guarantees not only a unique alignment for the reads matching the sequence, but also that no read with one or two base call errors or SNPs relative to the reference genome could be uniquely mismapped to this region. Mappability is important even if an aligner does not use heuristics and exhaustively enumerates read alignments. As demonstrated by Figure 1A, highly similar regions can produce uniquely mismapped reads as a result of genome variation and read errors in a way that no aligner can recognize (see Methods section for details).

If we restrict our attention to alignments in mappable regions, we ensure that the downstream analysis results are robust, even if the reference genome does not match the subject genome or the reads contain sequencing errors. Mappability is thus inversely related with sensitivity to genome variation and read errors.

Since RNA-seq reads may span multiple exons, the transcriptome contains additional k-mers beyond those found in the genome. To compute transcriptome mappability, we can align k-mers to the genome sequences crossing splice junctions. This transcriptome mappability scheme allows the computation of pseudogene expression levels using only reads uniquely aligned to mappable regions. Using these reliable reads, we compute pseudogene expression levels in units of Reads per Kilobase of Uniquely mappable transcript per Million reads (RPKUM). See the Methods section for a detailed description of the transcriptome mappability and RPKUM calculations.

We tested our RPKUM metric by comparing expression levels for protein coding genes computed with both RPKUM and RSEM [24], a commonly used transcript quantification method. We computed the mean expression level across the TCGA dataset for each protein-coding gene using both methods, then calculated the correlation between the expression levels from the two methods. The result showed good agreement between RPKUM and RSEM values (Spearman correlation > 0.85), indicating that RPKUM values provide a reliable method for quantifying expression levels.

An important question is whether RPKUM values computed from few mappable bases are trustworthy. To investigate the robustness of the RPKUM metric, we simulated RPKUM values by randomly sampling positions of genes that are completely mappable and then using these sampled bases as the only mappable bases of a gene in an RPKUM calculation. Genes spanning a wide range of expression levels from 1 to 200 RPKMs were used in the simulation. We performed the simulations with 500, 100, and 50 mappable bases per gene. RPKUM values computed from genes with as few as 50 simulated mappable bases showed very strong agreement with the true RPKM expression levels across the range of expression levels (ρ = 0.95). In addition, increasing the number of mappable bases slightly increases the correlation between RPKUM and RPKM levels (ρ = 0.97 for 100 mappable bases and ρ = 0.99 for 500 mappable bases).

**Figure 2:**
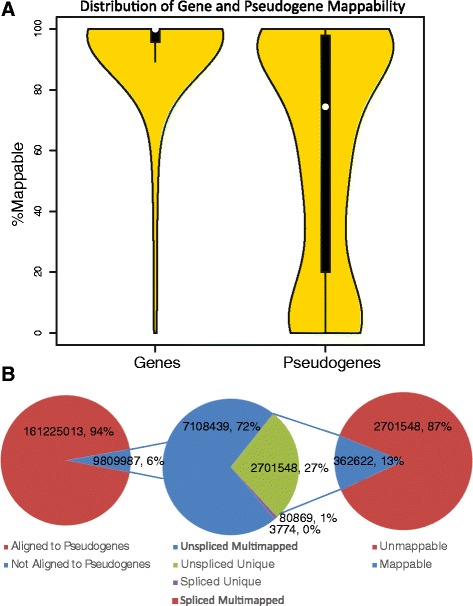
**Pseudogene mappability and read alignments. (A)** Violin plot showing the distribution of gene and pseudogene mappability as a percentage of gene length. The dot in the middle of each plot represents the median, and the black box is the interquartile range. **(B)** Pie charts showing how many reads are removed by mappability filtering. From left to right: Fraction of all aligned reads that map to pseudogenes; fraction of reads aligned to pseudogenes that are uniquely aligned; and fraction of reads uniquely aligned to pseudogenes that are also mappable.

Figure 2A shows the distribution of transcriptome mappability for protein coding genes and GENCODE v. 17 pseudogenes. As expected, pseudogenes are much less mappable than protein-coding genes; the median protein-coding gene mappability value is nearly 100% of gene length, and the vast majority of genes are almost completely mappable. In contrast, the median pseudogene mappability value is around 80% of pseudogene length. The distribution of pseudogene mappability is approximately bimodal, with peaks near 10% and 90%. A sizable fraction of pseudogenes are completely unmappable (2169 out of 14942). Nonetheless, the majority of pseudogenes possess a significant fraction of mappable bases and are thus accurately detectable using RPKUM expression levels.

As expected, restricting the set of reads aligned to pseudogenes to only those in mappable regions leads to a dramatic reduction in the number of reads (Figure 2B). On average, each sample contains nearly 10 million reads mapped to pseudogenes, but our filtering process leaves a set of just over 360,000 pseudogene reads per sample. The surviving reads comprise a high-confidence set that can be used to assess pseudogene transcription.

### High-confidence breast cancer pseudogene transcripts

**Figure 3:**
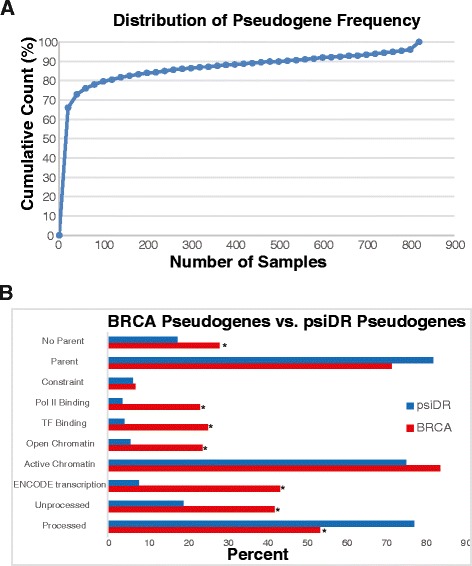
**Pseudogene occurrence in the TCGA breast cancer samples and overlap with ENCODE functional genomics annotations. (A)** Cumulative distribution function showing how many samples pseudogenes occur in. Approximately 65% of the 2,012 transcribed pseudogenes occur in fewer than 20 samples. Roughly 25% of the pseudogenes occur in at least 80 samples. **(B)** Bar chart comparing the set of 287 pseudogenes transcribed in breast cancer with the full psiDR v. 0 annotation set. The asterisks indicate categories that are significantly enriched in the set of 287 pseudogenes compared to the full set (*p* < 0.002, χ^2^ test).

Using the GENCODE v. 17 pseudogene annotations, we identified 2012 pseudogenes with evidence of transcription, defined as genes with at least 50 mappable bases, 50 reads, and 1 RPKUM in at least 1 sample (Additional file 1). The majority of these pseudogenes occurred in only a small number of samples (Figure 3A). However, a subset of the pseudogene transcripts occurs in a large number of samples, including 94 pseudogenes that are transcribed in over 95% (n = 780) of the samples. To investigate the pseudogenes that are most likely to play a role in cancer biology, we chose to focus the remainder of our analysis on pseudogenes that exhibited evidence of transcription in at least 10% (n = 80) of the samples; this set consists of 440 pseudogenes.

The GENCODE pseudogene decoration resource (psiDR v. 0), assembled from a recent genome-wide survey of pseudogenes using ENCODE data [3], provides useful information for an initial assessment of the transcriptional potential of our pseudogene set. Out of the set of 440 transcribed pseudogenes we identified, 287 pseudogenes are annotated in psiDR for a number of attributes, including pseudogene type, parent gene, transcription evidence, open chromatin, histone modifications that indicate activity, transcription factor binding, RNA polymerase II occupancy, and evolutionary constraint [3]. Although the functional genomics annotations come from the ENCODE cell lines, not from breast cancer tissue, they nonetheless serve as a reasonable starting point for assessing the transcriptional activity of the pseudogenes we identified.

Examining the collection of psiDR annotations for these 287 transcribed pseudogenes shows that they possess a number of properties that indicate transcriptional activity (Figure 3B). Nearly half (n = 125) of the 287 pseudogenes were reported by psiDR to be transcribed. The remainder (n = 162) represent potentially novel pseudogene transcripts not annotated in psiDR. The pseudogenes producing these unannotated transcripts show strong evidence of transcriptional activity. Compared to the full set of more than 11,000 pseudogenes annotated by psiDR, the set of 287 is significantly enriched for active chromatin, Pol II occupancy, and transcription factor binding (*p*<0.002,χ^2^test). In addition, 20 of these pseudogenes display fewer substitutions compared to chimp and mouse orthologs than expected by chance. Interestingly, duplicated and unitary pseudogenes are also enriched within the set of 287. This may be due in part to the fact that duplicated pseudogenes are thought to be more likely to possess upstream regulatory elements similar to those of the parent genes. Also, unitary pseudogenes are likely to be more mappable, and thus are easier to detect from short-read RNA-seq data. In short, the diverse data types from the ENCODE project provide strong support for the transcriptional activity of the pseudogenes that we have detected in breast cancer tissue.

It is worth noting that *PTENP1* and *KRASP1*, the two initial examples of pseudogene ceRNAs, are present (though at low levels) in the breast cancer samples we study here. Our method of computing RPKUM expression levels is thus capable of detecting these important pseudogenes, but their expression levels fall below the cutoff that we used to define our set of highly-expressed pseudogene transcripts, and therefore they were not considered for further analysis. The set of 748 breast-cancer pseudogene transcripts provided by Han et al. [22] does not contain *PTENP1* or *KRASP1*, confirming the low expression of these pseudogenes in breast cancer.

### Hierarchical clustering shows association with known cancer subtypes

**Figure 4:**
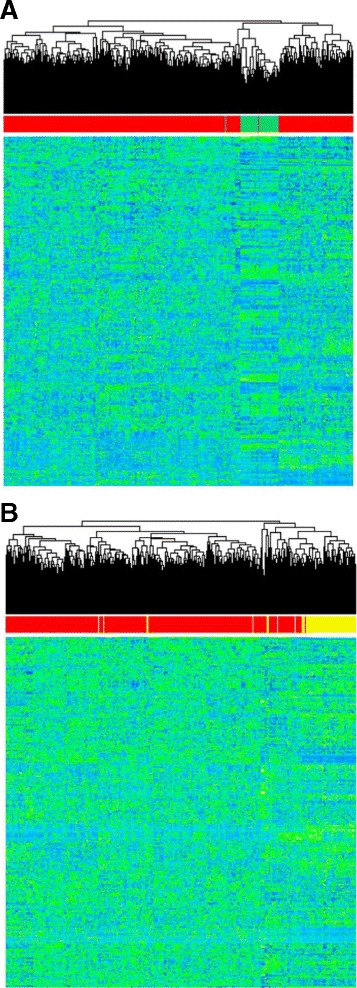
**Hierarchical clustering based on pseudogene expression shows pseudogene association with breast cancer subtypes. (A)** Heatmap showing pseudogene expression profiles in tumor and adjacent normal samples. High expression levels are shown in light green, and low expression levels are shown in light blue. Tumor samples are highlighted in red along the top of the plot; adjacent normal samples are highlighted in green. **(B)** Heatmap of pseudogene expression profiles in tumor samples. Samples belonging to the basal subtype are highlighted in yellow along the top of the plot.

The four molecular subtypes of breast cancer possess a number of distinguishing characteristics, including estrogen/progesterone receptor status, response to chemotherapy drugs, and gene expression profile [25]. A common method of studying the differences among these subtypes is to use unsupervised clustering techniques to group samples together based on their gene expression patterns. Unsupervised clustering using protein-coding genes results in four distinct clusters corresponding to the subtypes [25]. To investigate the relationship between pseudogene transcription and breast cancer disease state, we performed hierarchical clustering using the high-confidence set of 440 pseudogenes. Unsupervised clustering based solely on these pseudogene expression levels effectively separates tumor and normal samples (Figure 4A). However, since the normal samples are extracted from tumor adjacent breast tissue that contains a different cell type composition than the tumor itself, the ability to distinguish tumor from normal is likely due in large part to tissue specificity rather than tumor biology. Nonetheless, this result shows that pseudogene expression varies considerably between the cell types that make up the tumor and adjacent normal samples.

We also removed the adjacent normal samples and clustered solely on the tumor samples. As Figure 4B shows, the unsupervised clustering algorithm successfully separates the basal samples from the other subtypes. However, the pseudogene expression profiles for the luminal and Her2 subtypes are not sufficiently distinct to consistently separate samples from these subtypes. Basal tumors grow more rapidly and have significantly different histology than the other subtypes [25], and this may be why basal/luminal and basal/Her2 separation stands out more clearly than the luminal/Her2 separation. The fact that pseudogene expression alone can identify the basal subtype shows that pseudogene expression has a strong, non-random association with specific pathways and cellular environments. This suggests that previous findings, such as the pseudogene *ATP8A2*, which is more highly expressed in luminal compared to basal samples [4], are not isolated examples.

### Pseudogenes are differentially expressed among cancer subtypes

To identify the pseudogenes with the most strong subtype-specific expression profiles, we performed a multi-class differential expression analysis using the SAM tool [26]. This analysis yielded 309 pseudogenes with significant subtype-specific expression (FDR < 1%; Additional file 2). Several interesting pseudogenes are at the top of this list. For example, the second pseudogene on the list is ATP8A2-Ψ, a pseudogene that has been found to be upregulated in luminal subtypes and shown to induce tumor progression [4]. The expression profile found here reflects this pattern, showing strong upregulation in luminal samples compared to basal.

**Figure 5:**
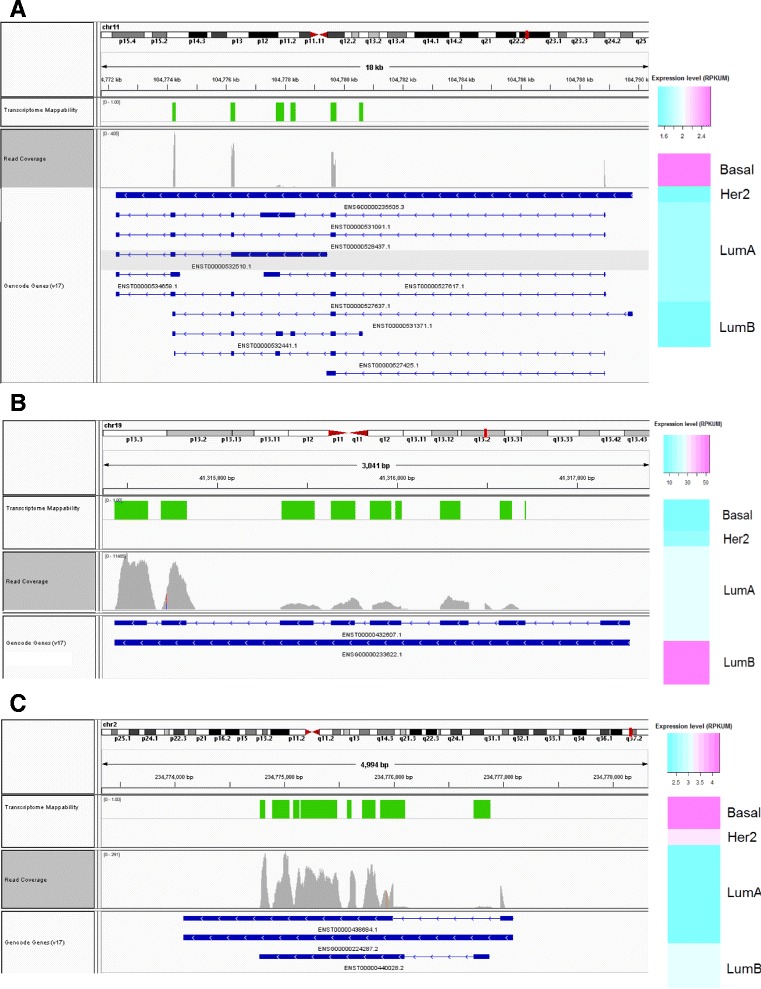
**Read coverage, mappability, and tumor expression profile for (A) CASP4 pseudogene, (B) CYP2F1 pseudogene, and (C) MSL3 pseudogene.** The light green bars in the top track of indicate regions that are mappable. Read coverage is shown by the height of the gray regions. Average expression level for each cancer subtype is shown to the right; pink indicates high expression, and light blue indicates low expression.

Three other interesting examples are shown in Figure 5. A pseudogene of CASP4, a member of the caspase family known to initiate apoptosis under certain conditions [27], is expressed at higher levels in basal samples and downregulated in luminal A samples (Figure 5A). Interestingly, the expression of the CASP4 pseudogene is lower in tumor samples than normal, which is the expression profile expected for a ceRNA that promotes CASP4 expression. Additionally, the CASP4 pseudogene was found to be transcribed in the ENCODE analysis [3]. Another interesting property of this unprocessed pseudogene is that it shows alternative splicing—there appear to be multiple isoforms represented in the reads covering the pseudogene locus. Intriguingly, our analysis of potential ceRNA interactions also indicated that the CASP4 pseudogene is positively correlated (ρ = 0.3) with expression of its parent gene and shares a miRNA target site for hsa-mir-203 (see next section for detailed summary of ceRNA investigation).

The CYP2F1 pseudogene is expressed at quite high levels compared to most pseudogenes in the dataset, and the average expression level in the luminal B subtype is nearly five times the average expression in the basal subtype. The pseudogene is a unitary pseudogene, with no clear parent protein-coding gene. However, it possesses strong sequence similarity with the cytochrome P450 family of genes. It was previously demonstrated that CYP2F1 is expressed in colorectal cancer and that expression in primary tumors correlated with corresponding metastatic tumors in lymph nodes [28]. Like the CASP4 pseudogene, the CYP2F1 pseudogene shows evidence for multiple isoforms.

A pseudogene of the MSL3 gene shows nearly twice the expression level in basal compared to luminal A (Figure 5C). The processed pseudogene was found to be transcribed in the ENCODE analysis. The MSL3 protein is thought to play a function in chromatin remodeling and transcriptional regulation, and it has been reported as part of a complex that is responsible for histone H4 lysine-16 acetylation [29]. Furthermore, expression of this pseudogene is correlated with the expression of its parent gene (ρ = 0.3), and it is predicted to share target sites for six different miRNAs (see next section for detailed summary of ceRNA investigation).

### Analysis incorporating miRNA and gene expression levels reveals pseudogenes with ceRNA potential

A common hypothesis about ceRNA interactions is that if transcript A sequesters miRNA C away from transcript B, the expression levels of A and B will be positively correlated, while both A and B will be negatively correlated with C. To assess the possibility that the transcribed pseudogenes identified may function as ceRNAs for their parent genes, we performed an analysis integrating miRNA target prediction with pseudogene, gene, and miRNA expression levels. The miRNA expression levels (Additional file 3) were computed from sample-paired TCGA small RNA-seq data using a previously described small RNA-seq analysis pipeline [30]. We computed expression levels for the parent genes of the pseudogenes using the same RPKUM method as for the pseudogenes.

Since pseudogenes are non-coding RNAs and are not densely bound by ribosomes, the vast majority of the transcribed region of a pseudogene is likely accessible for miRNA binding. However, if a pseudogene serves as a miRNA sponge for its parent gene, it is more likely that the shared miRNA binding site occurs in the 3′ UTR of the parent gene than in the coding region. In addition, using a restricted region for prediction somewhat ameliorates the lack of specificity common to miRNA target prediction algorithms [31]. We therefore chose to restrict our target prediction analysis to the portion of the pseudogene with sequence similarity to the 3′ UTR of the parent gene—what might be termed the “pseudo-3′ UTR”. During the process of performing miRNA target prediction on pseudogenes, we noticed that the GENCODE pseudogene annotations often did not span the pseudo-3′ UTR. Therefore, we used BLAST to identify the pseudo-3′ UTRs of pseudogenes by aligning the GENCODE annotation and surrounding genomic context with the annotated 3′ UTRs of the parent gene (see Methods section for details). TargetScan version 7 [32] was used to predict target sites for only the top 100 miRNAs expressed in the TCGA breast cancer dataset. This analysis revealed 177 transcribed pseudogenes that are predicted to share at least one miRNA target site with their corresponding parent genes.

**Figure 6:**
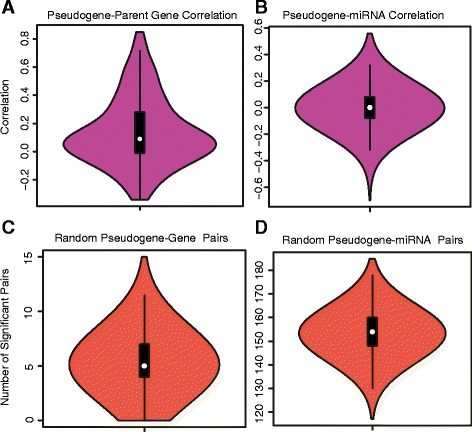
**Violin plots summarizing pseudogene-parent gene and pseudogene-miRNA pairwise correlations.** Correlations between **(A)** expressed pseudogenes and parent genes and **(B)** expressed pseudogenes and expressed miRNAs predicted to target them. Results of permutation analysis showing how many correlated pseudogene-parent gene pairs **(C)** and pseudogene-miRNA pairs **(D)** were found.

We computed Pearson correlation coefficients for each pseudogene-parent gene pair. As the plot in Figure 6 shows, the majority of pseudogene-parent gene pairs are uncorrelated. However, there is a positive skew to the distribution of correlations. To test whether the distribution of correlations differs significantly from expectation, we performed a permutation test. We constructed 5000 sets of gene-pseudogene pairs in which the genes and pseudogenes were randomly paired. The sets were of the same size as the set of pseudogene-parent gene pairs. For each random set, we computed the number of pairs with Pearson correlation above 0.3. In the 5000 random sets we generated, there were never more than 15 such pairs per set (Figure 6C). However, the set of correlations resulting from pairing pseudogenes and parent genes contains 55 pairs with correlation above 0.3. This indicates that the positive skew to the distribution of correlations shown in Figure 6A is very unlikely to be due to chance. We also tested an additional correlation threshold of 0.5 and observed a similar result, indicating that our findings are robust to the choice of correlation threshold.

We also computed the correlation between the expression level of each pseudogene and the miRNAs predicted to target it. The correlations observed for these pseudogene-miRNA pairs closely approximate a normal distribution, but show a slight negative trend (Figure 6B). A total of 180 pseudogene-miRNA pairs show strong negative correlation of less than −0.3. To test whether this number of pairs is significant, we approximated a null distribution of pseudogene-miRNA correlations using the same permutation method we applied to the pseudogene-parent gene pairs. Randomly shuffling the pseudogene-miRNA pairs to create 5000 random sets (Figure 6D) showed only 5 permutations with at least as many strongly anti-correlated pairs as we observed in the data, which corresponds to an empirical p-value of 0.001. This supports the conclusion that the extent of negative correlations observed in the data cannot be attributed solely to chance, and is likely due to genuine miRNA target repression.

Next we sought to identify the pseudogene-parent gene-miRNA triples with the strongest ceRNA potential. To do this, we first identified expressed miRNAs predicted to target both a pseudogene and its parent gene. For each such triple, we computed the correlation between pseudogene and parent gene, pseudogene and miRNA, and parent gene and miRNA (Additional file 4). We also computed p-values with Benjamini-Hochberg FDR correction for the miRNA correlations. In this way, we identified 17 pseudogene-gene pairs with strong ceRNA potential, which we defined as pseudogene-gene correlation greater than 0.3 and statistically significant miRNA anti-correlation.

Two of these pseudogenes stand out as especially interesting examples. A pseudogene of *GBP1* and its parent gene show statistically significant anti-correlation with hsa-mir-199a, which has been shown to regulate autophagy in breast cancer cells [33]. This pseudogene was also found to be transcribed in the ENCODE analysis [3]. The parent gene *GBP1* is known to be the mediator of the anti-proliferative effect of inflammatory cytokines in endothelial cells [34], and is implicated in several types of cancer according to GeneCards. In addition, the *GBP1* pseudogene shows strong positive correlation with the expression of its parent gene across the TCGA dataset (ρ = 0.82). Another interesting pseudogene is *SUZ12P1*. This pseudogene and its parent gene both show strong anti-correlation to hsa-mir-28. *SUZ12P1* also shows moderate positive correlation with its parent gene (ρ = 0.41). The parent gene, *SUZ12*, is a polycomb group protein and part of the PRC2/EED-EZH2 complex, an important epigenetic regulator that performs histone methylation [35]. This gene is also frequently translocated in endometrial stromal tumors, where it forms the *JAZF1-SUZ12* oncogene [36].

An interesting question is whether the genes that have pseudogenes with ceRNA potential are functionally related. To investigate this question, we performed a Gene Ontology (GO) term enrichment analysis using three different sets of parent genes. The sets of genes used were parent genes strongly correlated with a pseudogene, parent genes whose pseudogenes was strongly anti-correlated with a shared miRNA, and parent genes participating in a putative gene-pseudogene-miRNA ceRNA interaction as defined above. For each of these sets of parent genes, we used the GOrilla tool with default settings to look for GO terms enriched in the set compared to the background list of all parent genes. No significantly enriched GO terms were found for any of the 3 sets of interest, indicating that there is no clear functional relationship among the parent genes in the sets that we have identified.

## Discussion

**Figure 7:**
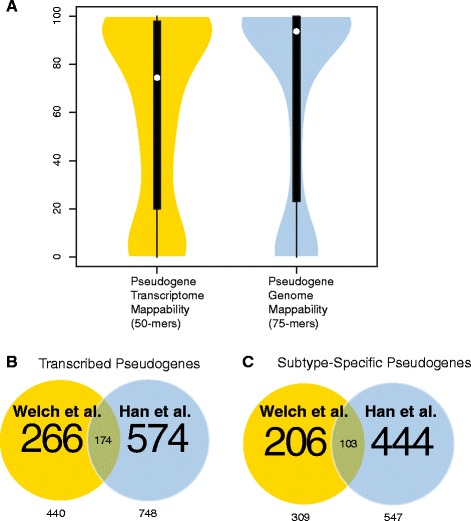
**Comparison with the results of Han et al. (A)** Violin plots showing the difference in pseudogene mappability when using 50-mers and accounting for splice junctions inserted in the genome (yellow) and 75-mers (blue). **(B)** Comparison with breast cancer pseudogene transcripts found by Han et al. **(C)** Comparison with breast cancer subtype-specific pseudogene transcripts found by Han et al.

The recent paper by Han et al. that investigated pseudogene expression in cancer [22] identified 748 pseudogenes transcribed in breast cancer, 547 of which showed subtype-specific expression. Although the results of Han et al. partially overlap with our own, our study is distinct in two key ways: (1) we investigate the ceRNA potential of pseudogenes transcribed in breast cancer, but Han et al. do not and (2) we use a more detailed method for measuring pseudogene transcription, designed to maximize specificity. In an effort to avoid the artifacts that plague pseudogene transcription detection, we designed our analysis to be as conservative as possible. Consequently, the set of pseudogenes detected by our method is somewhat smaller. However, our set of pseudogenes is not simply a subset of theirs. Out of the 440 pseudogenes we detect, only 174 were also found by Han et al. (Figure 7B). The remaining 266 represent novel pseudogene transcripts. In addition, 103 of the subtype-specific pseudogenes we identified overlap with the set of subtype-specific pseudogenes presented in Han et al. (Figure 7C).

To understand why our set of pseudogenes is substantively different from that of Han et al., we carefully analyzed how they computed pseudogene expression levels. They used 75-mers to compute mappability, and decided for each exon whether to include or exclude reads for the entire exon. One shortcoming of this approach is that it either includes or excludes reads for entire exons, rather than making decisions for individual reads. In our experience, small islands of similarity within an otherwise distinct exon are often enough to promote false positive read alignments. Conversely, small islands of distinct sequence within an exon can be used to detect the presence of pseudogene transcripts. As a result, our approach detected 266 pseudogenes with strong evidence of transcription that were overlooked in Han et al. [22]. Another limitation is that the analysis in [22] did not account for the presence of splice junctions inserted into the genome. Processed pseudogenes containing concatenated exons are a major source of error in pseudogene RNA-seq alignments because RNA-seq aligners sometimes prefer unspliced alignments to spliced, particularly in the presence of SNPs. However, genomic mappability as used in [22] cannot detect such artifacts.

A more serious problem is that although the RNA-seq reads from the TCGA BRCA data are 50 bases long, Han et al. use mappability based on 75-mers to decide which pseudogenes are mappable. Given that longer sequences are more likely to be distinct in the genome, this mismatch between read length and the k-mer size used to compute mappability means that an exon that appears completely mappable may nonetheless have many misaligned reads. Figure 7A shows the difference in mappability obtained from 75-mers without accounting for splice junctions inserted in the genome and 50-mers. In the first case, the median mappability as a percentage of gene length is 94%, but in the second case it is 74%. The use of 75-mers as in [22] rather than 50-mers results in a loss of specificity. Thus, it is possible that some of the pseudogenes transcripts detected in this way are not actually transcribed, but are simply read alignment artifacts.

In summary, two major differences between the approach of Han et al. and our own method for computing pseudogene expression explain the differing lists of pseudogenes that were obtained. First, Han et al. either kept or removed entire pseudogene exons, while we made the decision for each individual read; this explains why we detected some pseudogenes that they did not. Second, Han et al. used 75-mers to compute genome mappability, but we used 50-mers and accounted for processed pseudogenes containing splice junctions; consequently, our list of pseudogenes did not include some of theirs. We emphasized specificity in our algorithm in order to facilitate the identification of the highest confidence pseudogenes and candidate ceRNAs for further analysis. If the methods used to derive pseudogene expression levels do not properly account for misaligned reads, it is difficult to exclude the possibility that apparent pseudogene-based classification of subtypes are actually driven by improperly aligned reads from protein-coding genes with subtype-specific expression. Furthermore, such misaligned reads could bias toward stronger positive correlations between parent genes and pseudogenes.

In this paper, we undertook an initial investigation to address the important questions of how pervasive the pseudogene ceRNA mechanism is and how pseudogene transcription relates to breast cancer subtype. Careful scrutiny of RNA-seq evidence yielded a high-confidence set of pseudogene transcripts, a subset of which exhibit strong subtype-specific expression and are candidates for ceRNA function. Further experimental work is needed to examine these candidates; in particular, assays for miRNA binding and siRNA knockdown experiments can provide more conclusive evidence for ceRNA interactions in individual gene-pseudogene pairs. Follow-up studies are also needed to determine the nature of the relationship between pseudogene expression and subtype. Many of the subtype-specific pseudogene transcripts are likely passengers rather than drivers. However, some of these may play a role in the tumor progression of individual subtypes, as was demonstrated in the case of *ATP8A2-Ψ*.

The integration of pseudogene, gene, and miRNA expression data demonstrates that while not all pseudogenes may function as ceRNAs, the phenomenon is likely more pervasive than currently appreciated. One limitation of our approach is that ceRNA activity may not always be indicated by positive correlation between a pseudogene and its parent gene or negative correlation between a pseudogene and its targeting miRNA. For example, if the miRNA regulation of a pseudogene is very strong, leading to rapid and robust degradation of the pseudogene, this could produce a negative correlation between pseudogene and parent gene. Furthermore, it is well-known that regulatory network structures such as incoherent feed-forward loops can produce positive correlation between an mRNA and a targeting miRNA [37]. Even with this limitation, our results suggest that more pseudogenes than currently known likely function as ceRNAs, and more detailed experimental work is required to determine the physiological significance of this function.

## Methods

### Computing transcriptome mappability

A first approach to reliably studying pseudogene expression is to consider only reads that are assigned to a single location by an aligner. However, the confounding factors of SNPs, read errors and aligner heuristics can result in reads that are uniquely aligned to the wrong positions (Figure 1A). We refer to such reads as uniquely misaligned reads. Any conclusions drawn in the presence of uniquely misaligned reads in downstream analyses will be unreliable. In order to guard against this problem, we should distrust any reads for which there exist multiple possible alignments whose distance from the genome is less than some safety margin ε (Figure 1B). In such cases, there is sufficient ambiguity that we cannot rule out the possibility of unique misalignment.

To address the problem of read mismapping between genes and pseudogenes, we developed an approach based on the concept of mappability. Since RNA-seq reads may span multiple exons, the transcriptome contains additional *k* -mers beyond those found in the genome. In considering transcriptome *k* -mers, two cases arise that are particularly problematic for pseudogenes: processed pseudogenes with integrated splice junctions and duplicated pseudogenes that may have highly similar splice junctions to their parent genes. The former case is particularly problematic because RNA-seq aligners sometimes prefer direct alignments to spliced alignments, causing spuriously aligned reads to accumulate on processed pseudogenes. To compute transcriptome mappability, we consider *k* -mers from the genome and “synthetic regions” surrounding splice junctions (Figure 1D). The synthetic region around a splice junction is the concatenation of the immediately adjacent *k*–1 bases from donor and acceptor exons. These regions thus contain any *k* -mers that span annotated splice junctions. For a given genome G, transcriptome T (represented as *k* -mers from synthetic regions), position *i*, read length *k* and error tolerance ε, we define the mappability of position *i* as a Boolean quantity:

$$ M\left(G,T,i,k,\varepsilon \right)=\left\{{}_{1\kern1em \mathrm{other}\mathrm{wise}}^{0\kern1em i\mathrm{f}\ {G}_i \dots {G}_{i+k-1}\kern0.5em \mathrm{is}\ \mathrm{within}\ \mathrm{Hamming}\ \mathrm{distance}\ \varepsilon\ \mathrm{of}\ \mathrm{any}\ \mathrm{other}\ k\hbox{-} \mathrm{m}\mathrm{e}\mathrm{r}\ \mathrm{in}\ G\ \mathrm{or}\ T}\right. $$

### Finding transcribed pseudogenes

We filtered reads by requiring that either (1) the read has a unique, direct alignment to the genome starting at position *i* and this position is mappable or (2) the read has a unique, spliced alignment and the spliced *k* -mer to which the read is aligned occurs exactly once in the genome and transcriptome. We refer to reads surviving this filtering as “mappable reads”. Ensembl protein-coding gene annotations and GENCODE pseudogene v. 14 annotations were used to compute synthetic regions around splice junctions.

The number of mappable bases for each pseudogene was computed by constructing a “consensus pseudogene model” in which all annotated exons are merged into a nonredundant set of positions including all potentially transcribed regions from the gene model. We count a position within the resulting nonredundant set of transcript positions as mappable if either (1) the corresponding position in the genome is mappable or (2) a mappable spliced read occurs at that position.

Using the reliably mapped reads and mappable bases, compute pseudogene expression levels in units of Reads per Kilobase of Uniquely mappable transcript per Million reads (RPKUM):

$$ \mathrm{Expression}\ \mathrm{level}\ \mathrm{in}\ \mathrm{RPKUM} = \frac{{\mathrm{Mappable}\ \mathrm{reads}\ \mathrm{from}\ \mathrm{pseudogene} \times 10}^9}{\mathrm{Mappable}\ \mathrm{bases}\ \mathrm{in}\ \mathrm{pseudogene} \times \mathrm{total}\ \mathrm{reads}} $$

The justification for computing expression levels in units of RPKUM instead of RPKM is that reads aligned to unmappable regions are not considered in the expression level calculation, so counting the total number of bases in the transcript would underestimate the expression level. One limitation of the RPKUM metric is when the regions used to determine pseudogene transcription are disjoint from a transcript isoform. In such a case the RPKUM expression measurement does not include the expression of the unmappable isoform. Out of 14,943 pseudogenes annotated by GENCODE v.17, only 89 pseudogenes have one or more unmappable transcript isoform (defined as < 50 mappable bases). Only 17 of these occur in the set of 440 that we analyze in the paper, and of this set of 17, only 5 have parent genes.

Figure 1C summarizes our pipeline for computing pseudogene expression levels. Our approach improves on the strategy used in [3] and [4]. In [38] a method was proposed that, as ours, tries to avoid uniquely misaligned reads and also included a measure of mappability. However, the method developed in [38] applied only to processed pseudogenes and could not be used for duplicated pseudogenes. Our method also accounts for the possibility of reads that cross splice alignments in defining mappability.

### Hierarchical clustering and differential expression analysis

Tumor subtype classification was determined using the PAM50 score [39]. Unsupervised hierarchical clustering was performed using the R function hclust. Expression levels were log transformed and normalized using the R scale function before clustering. We first performed clustering using both tumor and adjacent normal samples. Next, we omitted the adjacent normal samples and clustered only the tumor samples. To determine which pseudogenes showed significant subtype-specific expression, we used the Significance Analysis of Microarrays R package (samr) [26]. This approach uses a nonparametric test based on the Kruskal-Wallis statistic to assess the evidence for rejecting the null hypothesis that the expression levels do not differ among subtypes. The multiclass differential expression option of the samr package was used.

### Prediction of miRNAs targeting pseudogenes and genes

Since pseudogenes are thought to be non-coding and thus not densely bound by ribosomes, the entire transcript can be targeted by miRNAs. Also, since pseudogenes are non-coding, 3′ UTRs are not annotated for pseudogenes. However, if a miRNA targets both a pseudogene and its parent gene, the shared miRNA binding site is likely to be located in the 3’ UTR of the parent gene and the corresponding “pseudo-3′ UTR” of the pseudogene. In order to be more conservative and in an effort to reduce the number of false positives arising from the lack of specificity in miRNA target prediction algorithms, we chose to restrict our analysis to the pseudo-3′ UTRs of pseudogenes; we therefore had to annotate these regions. Pseudo-3′ UTRs were annotated by BLAST alignment to the 3′ UTRs of the parent genes.

For each parent gene-pseudogene pair, we downloaded all annotated 3′ UTRs for the parent gene. Next, we extracted the pseudogene locus according to GENCODE and 10 kb of genomic context on either side of the pseudogene. BLAST was then used to align the parent gene 3′ UTRs against the pseudogene plus genomic context. The longest statistically significant alignment (based on the BLAST E-value) was taken to be the pseudo-3′ UTR. Target prediction was performed on pseudo-3′ UTRs and annotated gene 3′ UTRs using TargetScan version 7 [32]. Only miRNA target seeds from the top 100 expressed miRNAs by average expression level across the samples were used in the target prediction. Isomirs (mature miRNAs resulting from a shift in the annotated transcription start site of the same miRNA locus) were considered to be different miRNAs in this analysis. A miRNA was considered to be “shared” between a pseudogene and parent gene if TargetScan predicted that the miRNA could target both of them.

### Correlation with protein-coding gene and miRNA expression levels

We computed Pearson correlation coefficients on log-transformed gene and pseudogene expression levels using the parent gene annotations from the ENCODE pseudogene decoration resource (psiDR v. 0). To avoid detecting spurious correlations due to predominantly low expression, we required at least 20 samples in which gene and pseudogene are present at 1 RPKUM or greater. Gene-pseudogene pairs with fewer than 20 such samples were omitted from the analysis. We used the miRNA targeting predictions from TargetScan (see “Prediction of miRNAs targeting pseudogenes and genes”) to compute correlations between pseudogene and miRNA expression levels. Only the top 100 miRNAs by average expression level were used for this analysis. The pipeline described in Baran-Gale et al. [30] was used to compute miRNA expression levels from the TCGA small RNA-seq data. Correlations with miRNAs were assessed by computing p-values using a T-statistic for the null hypothesis that the correlation is no smaller than 0. False discovery rate correction using the method of Benjamini and Hochberg was performed with the R function p.adjust.

## Additional files

Additional file: 1.
**Pseudogenes transcribed in at least 1 breast cancer sample.**
Additional file: 2.
**Pseudogenes showing significant subtype-specific expression.**
Additional file: 3.
**Top 100 Expressed miRNAs.**
Additional file: 4.
**Pearson Correlation Coefficients for Pseudogenes, Parent Genes, and shared miRNAs.**

## Abbreviations

ceRNA: Competing endogenous RNA
miRNA: microRNA
TCGA: The cancer genome atlas
RPKUM: Reads per kilobase of unique transcript per million reads
